# The influence of contextual reward statistics on risk preference

**DOI:** 10.1016/j.neuroimage.2015.12.016

**Published:** 2016-03

**Authors:** Francesco Rigoli, Robb B. Rutledge, Peter Dayan, Raymond J. Dolan

**Affiliations:** a The Wellcome Trust Centre for Neuroimaging, UCL, 12 Queen Square, London WC1N 3BG, UK; b Max Planck UCL Centre for Computational Psychiatry and Ageing Research, London WC1B 5EH, UK; c Gatsby Computational Neuroscience Unit, UCL, 17 Queen Square, London WC1N 3AR, UK

## Abstract

Decision theories mandate that organisms should adjust their behaviour in the light of the contextual reward statistics. We tested this notion using a gambling choice task involving distinct contexts with different reward distributions. The best fitting model of subjects' behaviour indicated that the subjective values of options depended on several factors, including a baseline gambling propensity, a gambling preference dependent on reward amount, and a contextual reward adaptation factor. Combining this behavioural model with simultaneous functional magnetic resonance imaging we probed neural responses in three key regions linked to reward and value, namely ventral tegmental area/substantia nigra (VTA/SN), ventromedial prefrontal cortex (vmPFC) and ventral striatum (VST). We show that activity in the VTA/SN reflected contextual reward statistics to the extent that context affected behaviour, activity in the vmPFC represented a value difference between chosen and unchosen options while VST responses reflected a non-linear mapping between the actual objective rewards and their subjective value. The findings highlight a multifaceted basis for choice behaviour with distinct mappings between components of this behaviour and value sensitive brain regions.

## Highlights

• We use fMRI and a novel gambling paradigm to study the influence of the contextual reward statistics on decision making under risk
• Risk propensity is influenced by the distribution of rewards available in a given temporal context, an effect that relates to activity in ventral tegmental area/substantial nigra
• ventral striatum encodes elements of subjective, rather than objective, value during decision making under risk
• activity in ventromedial prefrontal cortex reflects the subjective value difference between chosen and unchosen option and not the average value across options

## Introduction

Context dramatically affects value-based choice (e.g., Huber, J., et al., 1982, Kahneman, D. and Tversky, A., 1979, Ludvig, E. A., et al., 2013, Stewart, N., et al., 2003, Tversky, A. and Shafir, E., 1992). A striking example is the so-called framing effect, in which risky options are preferred more when choices are framed in terms of losses rather than gains (Kahneman and Tversky 1979). Though contextual effects have been extensively described, we know little about the mechanisms through which contextual representations arise and contribute to decision making. One possibility is that prevailing contextual reward statistics influence the mapping from objective to subjective values and in this way affect choice. There are two competing hypotheses about how this might occur. One is that the contextual statistics of reward induce a reference point relative to which values are rescaled (Ludvig, E. A., et al., 2013, Stewart, N., et al., 2003, Stewart, N., et al., 2006). This predicts that, for example, the same dish is likely to be evaluated as being worse in a good restaurant than in a bad one. An alternative possibility derives from a Bayesian perspective that proposes objective reward values are integrated with prior subjective value expectations arising from a prevailing contextual reward distribution (Seymour and McClure 2008). Posterior subjective values would be hence estimated in such a way that they increase/decrease in contexts characterized by larger/smaller reward distributions. This makes an opposite prediction that the same dish will be evaluated as being better in a good restaurant than a bad one.

**Fig. 1:**
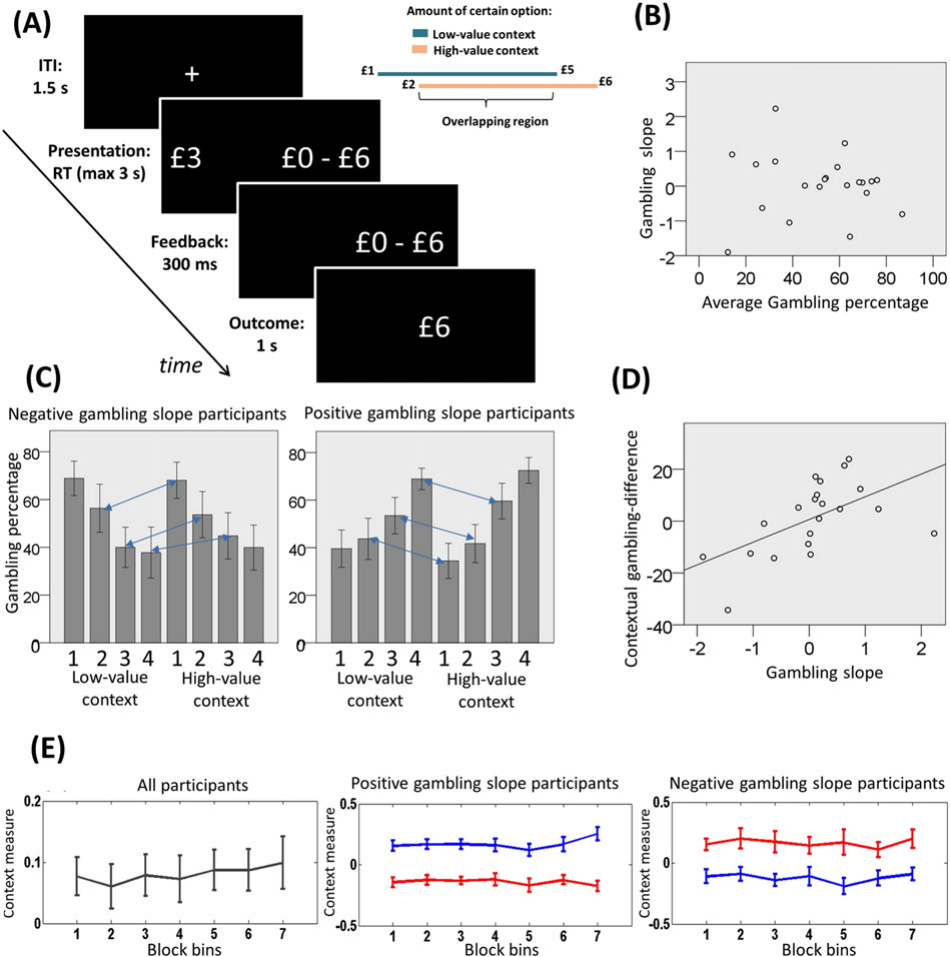
A: Experimental paradigm. Participants repeatedly made choices between certain gains (on the left in the example) and gambles (on the right in the example) associated with a 50% probability of either double the certain gain or zero. After a choice, the unchosen option disappeared and 300 ms later the trial outcome was shown for 1 s. The intertrial interval (ITI) was 1.5 s. At the end of the experiment, a single randomly chosen outcome was paid out to participants. B: Relationship between average gambling percentage (x-axis) and gambling slope (y-axis). This relationship was not significant (r(21) = − 0.06, p = 0.78, non-significant). Note that the gambling slope corresponds to the individual effect (i.e., the slope of a logistic regression parameter) of monetary amount on gambling, thus positive and negative gambling slopes correspond to increased gambling preference with increasing and decreasing amounts, respectively. A distribution of subjects (represented as dots) with positive and negative slopes is evident. C: Gambling percentage for different monetary amounts (grouped in 4 increasing magnitude bins: [1 2 3 4]) for each context (low and high). Participants are split in two groups based on their gambling slope (negative gambling slope: n = 9; positive gambling slope: n = 12). Blue arrows connect equivalent amounts presented in the two contexts. Consistent with a contextual normalization effect, subjects who gambled more with increasing amounts also gambled more when equivalent choices were relatively larger, that is in the low-value context. By contrast, subjects who gambled more with decreasing amounts also gambled more when equivalent choices were relatively smaller, that is in the high-value context. D: Relationship between gambling slope (x-axis) and contextual gambling difference for overlapping amounts (y-axis), corresponding to the gambling percentage in low-value minus high-value context for equal amounts (r(21) = 0.56, p = 0.008). E: Analysis of the evolution of the context effect over time. Blocks are separated into 7 bins. Values labelled as “context measure” represent an index of the context effect (see main text to see how this is obtained). Lines represent average across subjects and error bars represent standard error. The left panel combines all participants and shows that, after bins were aggregated in two sets (without considering the fourth bin), the values of the first three bins were overall not different from the values of the last three bins (t(20) = − 1.02; p = 0.319). Also, the value of the first bin was not significantly different from the value of the last bin (t(20) = − 0.758; p = 0.457) and was significantly larger than zero (t(20) = 2.46, p = 0.023). These data indicate that a context effect emerged from the very start of a new context presentation and remained stable across the duration of the block. On the middle and right panels, lines represent the risk preference for overlapping choices in the two contexts. Red and blue lines are for high- and low-value context, respectively. Participants are separated into two groups depending on whether they have a positive (middle panel) or negative (right panel) gambling slope.

We investigated contextual effects on choice by focusing on decision-making under risk. We designed a paradigm wherein subjects repeatedly chose between a sure amount of money (called the trial monetary amount), that varied trial-by-trial, and a gamble associated with an equal probability of obtaining either double the sure amount or zero (Fig. 1A). The trial outcome was displayed after each choice and one randomly selected outcome was paid out to participants at the end of the experiment. Crucially, trials were arranged in blocks each associated with one of two subtly different gambling contexts involving specific, but partially overlapping, distributions of monetary amounts. A high-value context involved monetary amounts drawn uniformly from £2–£6, and a low-value context involved monetary amounts drawn uniformly from £1–£5. In terms of contextual adaptation, for choices that are objectively equivalent across contexts, the rescaling hypothesis predicts larger subjective values (inferred from choice behaviour) in a low-value context (Ludvig, E. A., et al., 2013, Stewart, N., et al., 2003, Stewart, N., et al., 2006), whereas the Bayesian hypothesis predicts larger subjective values in a high-value context (Seymour and McClure 2008). Note that, since we did not aim to dissociate the effect of average monetary amount and variance of individual gambles on risky choice, we used simplified options in which these covaried perfectly.

A main goal was to investigate the relationship between behavioural and neural contextual adaptation effects. It is well-established that response in dopaminergic ventral tegmental area/substantia nigra (VTA/SN) and ventral striatum (VST) reflect a reward prediction error (RPE) signal (D'Ardenne, K., et al., 2008, Lak, A., et al., 2014, Niv, Y., et al., 2012, O'Doherty, J. P., et al., 2003, O'Doherty, J., et al., 2004, Park, S. Q., et al., 2012, Schultz, W., et al., 1997, Stauffer, W. R., et al., 2014, Tobler, P. N., et al., 2005), and evidence indicates that such a RPE signal adapts to contextual reward availability (Louie, K. and Glimcher, P. W., 2012, Park, S. Q., et al., 2012, Rangel, A. and Clithero, J. A., 2012, Tobler, P. N., et al., 2005). However, whether such neural adaptation impacts choice behaviour remains to be tested, and there is controversy surrounding this issue (Louie, K. and Glimcher, P. W., 2012, Padoa-Schioppa, C. and Rustichini, A., 2014, Rangel, A. and Clithero, J. A., 2012). We were specifically interested in probing linkages between behavioural and neural response adaptation in VTA/SN and VST. Thus we used functional magnetic resonance imaging (fMRI) to measure neural activity during simultaneous task performance.

We also planned to exploit individual differences in choice preference to investigate the neural mechanisms underlying risky decision-making. Previous observations have shown that the degree of behavioural loss aversion is connected with the individual strength of VST activation for gains compared to losses (Tom et al. 2007), while the degree of behavioural risk preference is connected with a VST response to risky compared to non-risky options associated with equal average amount (Niv et al. 2012). Additionally, it has been reported that the VST response to reward probability follows a non-linear probability weighting function akin to that proposed within Prospect Theory (Hsu et al. 2009). However, whether the VST response to choice options with different levels of reward amount/variance reflects a non-linear subjective value function akin to that predicted by economic theories remains unclear (D'Acremont, M. and Bossaerts, P., 2008, Kahneman, D. and Tversky, A., 1979, Schultz, W., et al., 2008, von Neumann, J. and Morgenstern, O, 1944). We explored this question by exploiting the variability in participants' risk preference as a function of the monetary amounts presented.

Ventromedial prefrontal cortex (vmPFC; as distinct from the adjacent lateral orbitofrontal cortex which has been the topic of single-cell recording studies in non-human primates; Rushworth, M. F., et al., 2011, Strait, C. E., et al., 2014) plays a key role in decision-making by representing aspects of value (Bartra, O., et al., 2013, Blair, K., et al., 2006, Boorman, E. D., et al., 2009, FitzGerald, T. H., et al., 2009, Plassmann, H., et al., 2007, Tom, S. M., et al., 2007). However, the specific nature of signalling in this region is an object of ongoing debate (Rushworth et al. 2011). Recent findings fit with the idea that, at the time of a decision, activity in vmPFC relates to the subjective value difference between chosen and unchosen options (Boorman, E. D., et al., 2009, FitzGerald, T. H., et al., 2009, Hunt, L. T., et al., 2012, Strait, C. E., et al., 2014). However, other data support the hypothesis that a response in this region relates to the average subjective value available across options (Blair et al. 2006), possibly at an earlier time point during the decision phase (Hunt et al. 2012). One common problem is that subjective value differences and average values are highly correlated in many paradigms. However, in our task, these quantities are decorrelated across subjects, since the average subjective value depends largely on the trial monetary amount, whereas the subjective value difference depends upon individual gambling preferences. We exploited this feature to test the precise value correlates of the blood-oxygen-level-dependent (BOLD) signal in the vmPFC.

Substantial evidence implicates the anterior insula in representing stimulus salience as activity in this region can increase for both reward and punishment (Bartra et al. 2013). In addition, the insula response is correlated with risk-related variables, such as the entropy of an expected outcome (Critchley, H. D., et al., 2001, Preuschoff, K., et al., 2008, Rudorf, S., et al., 2012). However, despite these findings it remains unclear whether such a signal is associated with risk-taking behaviour per se. For example, a report of an insula response linked to switching from choosing risky options to choosing safer options hints at an association with risk aversion (Kuhnen and Knutson 2005). However, in this study the reinforcement history was relevant for behaviour and other studies also point to a role for the insula in learning from negative experiences (Palminteri et al. 2012), consistent with the possibility that insula might be activated by negative outcomes after risky choices. Hence it remains unclear whether reported effects are explained by risk aversion or learning. To address this issue, we investigated whether insula activity predicts choice of the gamble or of the safe option using a paradigm where learning is unnecessary.

## Methods

### Participants

Twenty-five healthy right-handed adults participated in the experiment. Three subjects were excluded from analyses because they missed more than 50 trials (see below). One subject was excluded because he decided to end the scanning session. Thus, the experimental sample included 21 subjects (13 females and 8 males, aged 20–40, mean age 27). On average, two missed trials were observed for these participants (range, 0–10). The study was approved by the University College of London Research Ethics Committee.

### Experimental paradigm and procedure

Inside the MRI scanner, participants performed a computer-based decision-making task lasting approximately 40 min (Fig. 1A). On each trial, participants chose between a certain monetary amount, which changed trial-by-trial, and a gamble whose prospects were always zero and double the certain amount, each with equal probability. Therefore, in every trial the certain option and the gamble always had equal average amount. Participants completed 4 blocks (140 trials each). In each block, the certain amount was randomly drawn from a uniform distribution (with 10 p steps): for two blocks (low-value context) the range was £1–£5; for the other two blocks (high-value context) it was £2–£6. Blocks were interleaved with 10 s breaks. Before each block, a panel showed the upcoming amount distribution. Block order was counterbalanced across subjects. After a 1.5 s intertrial interval, options were displayed on the left and right sides of the screen. Participants chose the left or right option by pressing the corresponding button of a keypad. Immediately after the choice was made, the unchosen option disappeared for 300 ms and next the amount gained was displayed for 1 s. Participants had 3 s to make their choices; otherwise the statement “too late” appeared and they received an outcome of zero. Positions of the certain and risky options were pseudorandomized, as well as outcomes of the gamble. At the end of the experiment, one outcome was randomly selected among those received and added to an initial participation payment of £17.

Participants were tested at the Wellcome Trust Centre for Neuroimaging at the University College London. Before scanning, they were fully instructed about the task and practised for up to 20 unpaid trials. Inside the scanner, participants performed the task in two separate sessions, each consisting of one low-value and one high-value context block, followed by a 12 minute structural scan. After scanning, participants were debriefed and informed about their total remuneration.

### Computational model of choice behaviour

We characterized choice behaviour by fitting a mean-variance return model that computed subjective values consistent with individual choices. Note that the experimental design precluded distinguishing such a model from an expected non-linear utility account, given the perfect correlation between the trial monetary amount and the variance. If the trial monetary amount (i.e., the certain reward) was A, then the value of the certain option was *V*_CERT_(*A*) = *A* - *χτ*, where *χ* is an indicator of it being the low-value (*χ* = 0)  or high-value context (*χ* = 1), and τ implements (subtractive) normalization of the certain amount associated with the latter context. This implies that the mean and variance of the gamble are *A* - *χτ* and (*A* - *χτ*)^2^ respectively, making the value of the gambling option be *V*_GAMB_(*A*) = *A* - *χτ* + α (*A* - *χτ*)^2^ + *μ* where α determines whether (α > 0) or not (α < 0) reward variance is attractive, and μ represents a gambling bias parameter. According to the model, the probability of choosing the gamble is given by a sigmoidal choice rule *σ*(*V*_GAMB_(*A*) - *V*_CERT_(*A*)) = 1/(1 + exp (-*V*_GAMB_(*A*) + *V*_CERT_(*A*))).

### fMRI scanning and analysis

The task was programmed with the Cogent toolbox (Wellcome Trust Centre for Neuroimaging) in Matlab. Visual stimuli were back projected onto a translucent screen positioned behind the bore of the magnet and viewed via an angled mirror. Blood oxygenation level dependent (BOLD) contrast functional images were acquired with echo-planar T2*-weighted (EPI) imaging using a Siemens Trio 3-Tesla MR system with a 32 channel head coil. To obtain more data in our regions of interest (ROIs), a partial volume of the ventral part of the brain was recorded. Each image volume consisted of 25 interleaved 3-mm-thick sagittal slices (inplane resolution = 3 × 3 mm; time to echo = 30 ms; repetition time = 1.75 s). The first six volumes acquired were discarded to allow for T1 equilibration effects. T1-weighted structural images were acquired at a 1 × 1 × 1 mm resolution. Functional MRI data were analysed using Statistical Parametric Mapping (SPM) version 8 (Wellcome Trust Centre for Neuroimaging). Data preprocessing included spatial realignment, unwarping using individual field maps, slice timing correction, normalization and smoothing. Specifically, functional volumes were realigned to the mean volume, were spatially normalized to the standard Montreal Neurological Institute (MNI) template with a 3 × 3 × 3 voxel size, and were smoothed with 8 mm Gaussian kernel. High-pass filtering with a cutoff of 128 s and AR(1)-model were applied. All general linear models (GLMs) included 6 movement regressors of no interest in addition to the regressors described below. Each GLM was estimated separately for each half of each of the two sessions of the task (corresponding to one single presentation of a context).

We estimated a GLM including a stick function regressor at option presentation modulated by (i) the average subjective value across options, (ii) the subjective value difference between chosen and unchosen option, (iii) a binary variable indicating whether the gamble or the certain option was chosen. The subjective value difference between chosen and unchosen option and the subjective value of the chosen option were estimated with the computational model of choice behaviour described above. The GLM included also one stick function regressor at outcome presentation modulated by RPE, computed as the difference between the subjective value of the obtained outcome minus the subjective value of the chosen option. Thus, RPEs were equivalent to zero for choices of the certain option and had positive or negative values for choices of the gamble. Since large average subjective values were associated both with very positive and very negative RPEs, the parametric modulators included in the GLM at option presentation were uncorrelated with RPEs. To obtain such decorrelation we included at outcome receipt the RPE instead of including separately the subjective value of the chosen option and the subjective value of the outcome. We also estimated another GLM where, for each context, trials were grouped in four bins on the basis of the certain amount, resulting in 8 bins in total (4 bins for each context). This GLM included separate stick function regressors at option presentation associated with each bin, plus a stick function regressor modulated by RPE at outcome time.

For each GLM contrasts of interest were computed subject by subject, and used for second-level one-sample t-tests and regressions across subjects. Predictors of regression models were the individual parameters estimated with the behavioural computational model. The regression model of the neural activation at option presentation for all amounts in the low minus high-value context included as predictor the context coefficient τ and binary variables encoding the individual block order condition. Statistical tests focused on the following ROIs: VST, VTA/SN, vmPFC and anterior insula. For VST and VTA/SN we used bilateral anatomical masks (defined manually using the software MRIcro and the mean structural image for the group) and for vmPFC and anterior insula we used 10 mm spheres centred on coordinates from a meta-analysis (Bartra et al. 2013). For hypothesis testing, we adopted voxel-wise Small Volume Correction (SVC) with a p < 0.05 Family Wise Error used as significance criterion.

## Results

### Behaviour

Across participants, average gambling percentage did not differ from 50% (mean = 51.5; SD = 21.27; t(20) = 0.32, p = 0.75; two-tailed p < 0.05 is used as the significance criterion for all behavioural tests). Given the fixed relationship between the gamble and the certain gain, the only independent measure varying trial-by-trial was the objective average monetary amount (called the trial monetary amount), which was equal for both options on a trial. We assessed the impact of this variable in a logistic regression model of gambling probability, finding that its influence over choice was statistically significant in 16 (half with positive and half with negative effect of trial amount on gambling) of 21 subjects, but with a direction that varied across participants (t(20) = 0.60, p = 0.55). We found no significant influence of other possible measures on choice (see supplementary data). There was no significant correlation between the individual effect of trial monetary amount (i.e., the slope parameter of the logistic regression model) and the average gambling percentage (r(21) = − 0.06, p = 0.78; Fig. 1B). This suggests that two partially independent factors contribute to risk attitude, namely a baseline gambling tendency and an increasing preference towards gambling for smaller or larger amounts.

We next tested for a context effect. We found no difference between contexts in the overall gambling percentage (t(20) = 0.35; p = 0.73), or in gambling percentage for overlapping amounts (t(20) = 0.37; p = 0.72). However, across individuals a positive correlation was evident between (i) the differential gambling percentage for overlapping amounts (i.e., the gambling percentage in low-value minus high-value context), and (ii) the effect of amount on gambling percentage (i.e., the slope parameter estimated in a logistic regression; r(21) = 0.56, p = 0.008; Figs. 1C–D). Based on this observation, we multiplied the effect of trial amount on gambling percentage with the differential percentage for overlapping amounts and found this variable was significantly positive across participants (t(20) = 2.55; p = 0.019). Put simply this shows that an influence of context interacted with an individual's propensity to gamble more with large or small amounts. In other words, participants who risked more with increasing amounts gambled more when equivalent choices were larger compared to the context, whereas participants who risked more with decreasing amounts gambled more when equivalent choices were smaller compared to the context. These findings indicate that subjective values of equivalent choices are larger in a low-value context (and vice-versa), and provide support for a contextual rescaling hypothesis (Stewart, N., et al., 2003, Stewart, N., et al., 2006) but no support for a contextual assimilation hypothesis (Seymour and McClure 2008).

In our task, prior to a new block a panel indicated to subjects the range of trial monetary amounts (i.e., £1–£5 and £2–£6 for the low- and high-value context respectively; see also methods), a procedure designed to induce an immediate contextual adaptation. To examine the impact of this manipulation we explored the temporal evolution of the context effect by dividing into seven bins (20 trials each) the blocks in which a shift of context occurred, and then averaging the gambling proportions for overlapping trial amounts (after subtracting these to the final bins of the previous blocks). The middle and right panels of Fig. 1E show the gambling proportion separately in subjects who preferred to gamble more with larger or smaller monetary amounts respectively, distinguishing between high- and low-value contexts; the left panel of Fig. 1E aggregates all participants and describes the evolution of the context effect as the difference in gambling proportions for low minus high-value contexts for participants who gambled more with larger amounts, and vice-versa for participants who gambled more with smaller amounts. It is apparent that there is no systematic change across blocks on average. We also confirmed this by showing that the mean value in bins 1–3 did not differ significantly from the mean value in bins 5–7 (t(20) = − 1.02; p = 0.319). Furthermore, the value of the first bin was not significantly different from the value of the last bin (t(20) = − 0.758; p = 0.457) and was significantly larger than zero (t(20) = 2.46, p = 0.023). These data support a view that the reported context effect emerged at the very beginning of a new context presentation and remained stable for the entire duration of the block.

We next characterized choice behaviour by fitting a mean-variance return model that computed subjective values consistent with individual choices (see Methods). Using BIC scores for comparison, the best fitting model included a gambling bias parameter μ, a parameter α indicating the preference for gambling with large (α > 0) or small (α < 0) amounts/variances, and a parameter τ implementing a contextual adaptation. This model performed better than more complex models including those whose parameters were estimated independently in each of the two contexts or in each half of the task, and simpler models in which some parameters were fixed (Table 1; see also supplementary data). The context coefficient τ was significantly positive across participants (Wilcoxon signed-rank Z(21) = 2.03, p = 0.042), consistent with the proposal that subjects' choices on average were affected by a reward adaptation mechanism. The value function coefficient α correlated with the individual effect of amount on gambling percentage (r(21) = 0.97, p < 0.001).

Note that normalization in the model is subtractive. We also considered divisive normalization such that, in the high-value context, the parameter τ was divided by the relevant amounts (parameters μ and α were also included in the divisive normalization model). The subtractive normalization model was preferred to the divisive normalization model by Bayesian model comparison (BIC = 12665 vs 12840).

Finally, to ascertain that the model can reproduce the behavioural results, we simulated choice data for each subject using their individual fitted parameters, and re-ran the model-free analysis on that simulated data. Consistent with our empirical results, in the simulated data we found (i) no correlation between the effect of trial amount on gambling and the average gambling proportion (r(21) = − 0.12, p = 0.60), and (ii) a correlation between the effect of trial amount on gambling and the difference in gambling for overlapping amounts across contexts (r(21) = 0.64, p = 0.001). By contrast, when we simulated a simpler model that omitted a baseline gambling parameter μ (based on parameters fitted for this model), we found, contrary to the empirical data, that the effect of trial amount on gambling and the average gambling proportion were correlated (r(21) = 0.71, p < 0.001). When we simulated a simpler model with no context parameter τ (based on parameters fitted for this model), again contrary to the empirical data, the effect of trial amount on gambling and the difference in gambling for overlapping amounts across contexts were not correlated (r(21) = 0.09, p = 0.70). Overall, the analysis on simulated data shows that the preferred model according to the BIC score is consistent with the behavioural results and simpler models do not reproduce the main features of the data.

### Neuroimaging

We used our computational model of choice behaviour to probe the neural processes underlying risk-based decision-making and its modulation by context. First, we investigated responses in brain regions involved in value-based choice. The fact that risk preferences varied across individuals allowed us to isolate the contribution of different value-related variables that many previous paradigms leave correlated. Thus, by using the behavioural computational model and individual parameters, for each trial we could estimate the average subjective value across options, the value of the chosen minus unchosen option, and a binary variable indicating whether the gamble or the certain option was selected. Though these variables showed significant within-subject correlations in many participants, their relationship was not systematic across subjects (see Table 2), allowing us to test their specific impact on brain activity (see supplementary data for further analyses). We used a GLM including a stick function regressor at option presentation modulated by (i) the subjective value averaged across the two options, (ii) the subjective value of the chosen minus unchosen option and (iii) a binary variable indicating choice of the gamble or choice of the certain option.

**Fig. 2:**
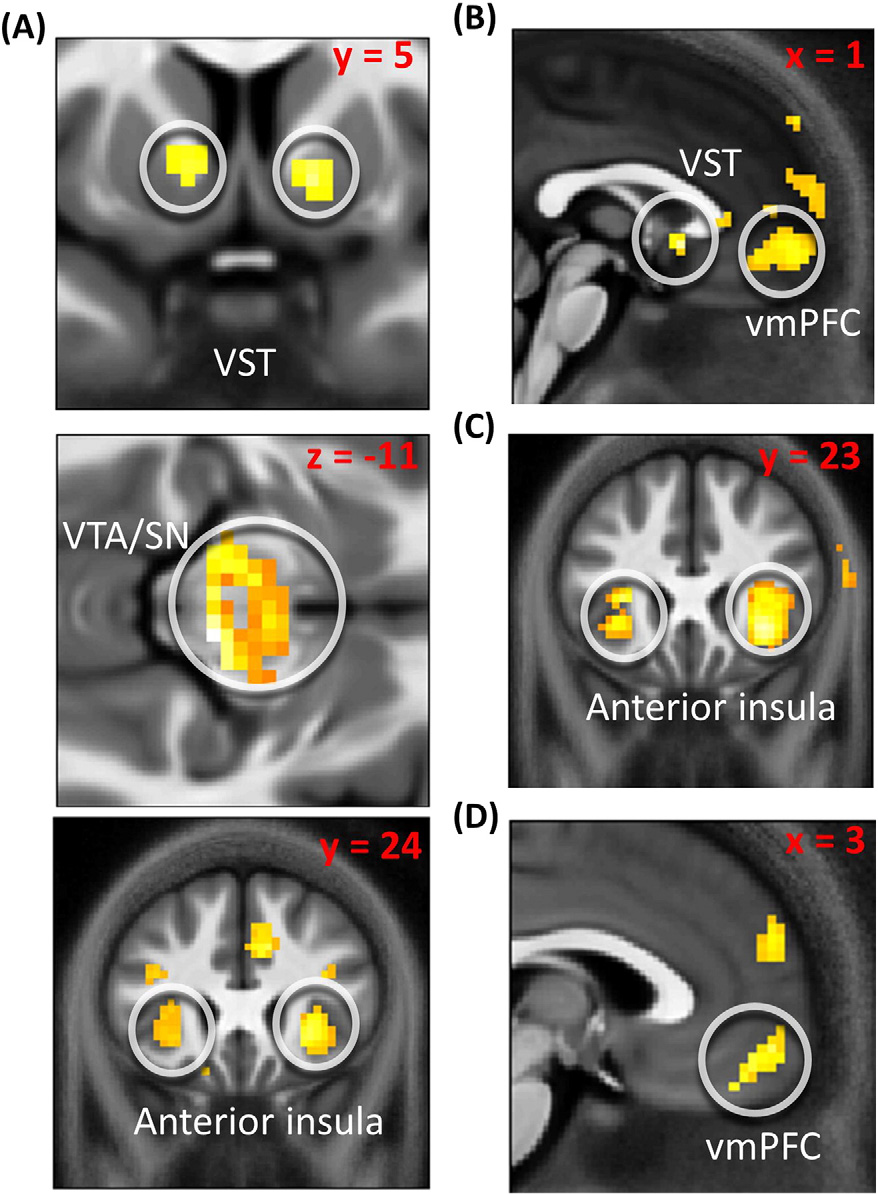
A: Brain activation correlating with average subjective value across options in (from top to bottom) VST (right: 9, 11, − 2; Z = 2.68, p = 0.049 SVC; left: -9, 11, − 2; Z = 3.00, p = 0.021 SVC), VTA/SN (right: 6, − 22, − 11; Z = 3.20, p = 0.010 SVC; left: -9, − 19, − 11; Z = 3.26, p = 0.009 SVC) and anterior insula (right: 30, 26, − 2; Z = 3.72, p = 0.007 SVC; left: -30, 29, 1; Z = 3.26, p = 0.025 SVC). B: Brain activation correlating with the value of the chosen option minus the value of the unchosen option in vmPFC (0, 56, − 5; Z = 2.92, p = 0.042 SVC) and right VST (3, 11, − 5; Z = 2.76, p = 0.033 SVC). C: Increased response for gambling choices compared to certain option choices in right anterior insula (33, 23, − 5; Z = 3.02, p = 0.033 SVC; the effect in left insula did not survive correction for multiple comparison). D: Increased response for certain option choices compared to gambling choices in vmPFC (3, 56, − 11; Z = 3.09; p = 0.045 SVC). These results were obtained using a GLM including a regressor at option presentation modulated by the average subjective value across options, the subjective value difference for the chosen minus unchosen option, and a binary variable indicating whether the gamble or the certain option was chosen. These variables were uncorrelated across participants, allowing us to separate their specific impact on brain activity.

Activity correlating with average value across options was seen in bilateral VST (right: 9, 11, − 2; Z = 2.68, p = 0.049 SVC; left: − 9, 11, − 2; Z = 3.00, p = 0.021 SVC; Fig. 2A; Montreal Neurological Institute coordinate system is used), bilateral anterior insula (right: 30, 26, − 2; Z = 3.72, p = 0.007 SVC; left: − 30, 29, 1; Z = 3.26, p = 0.025 SVC; Fig. 2A), and bilateral VTA/SN (right: 6, − 22, − 11; Z = 3.20, p = 0.010 SVC; left: − 9, − 19, − 11; Z = 3.26, p = 0.009 SVC; Fig. 2A), but not in vmPFC even at p < 0.05 uncorrected. It has been reported that vmPFC is activated maximally at the start of a task, when subjects are not over-trained (Hunt et al. 2012). To explore the possibility that vmPFC encodes average subjective value across options at the beginning of our task alone, we tested for an association between this variable and a vmPFC response separately for each of four blocks as a function of time. For no block (including the first one in the sequence) could we find a relationship between this variable and vmPFC activation, even at p < 0.05 uncorrected. Instead we found that the value of the chosen minus unchosen option correlated with activity in both vmPFC (0, 56, − 5; Z = 2.92, p = 0.042 SVC; Fig. 2B) and right VST (3, 11, − 5; Z = 2.76, p = 0.033 SVC; Fig. 2B). When comparing risky against non-risky choices, right anterior insula was more activated for the former (33, 23, − 5; Z = 3.02, p = 0.033 SVC; Fig. 2C) whereas vmPFC was more activated for the latter (3, 56, − 11; Z = 3.09; p = 0.045 SVC; Fig. 2D). We re-ran this analysis including reaction times (RTs) as an additional predictor and obtained similar results.

**Fig. 3:**
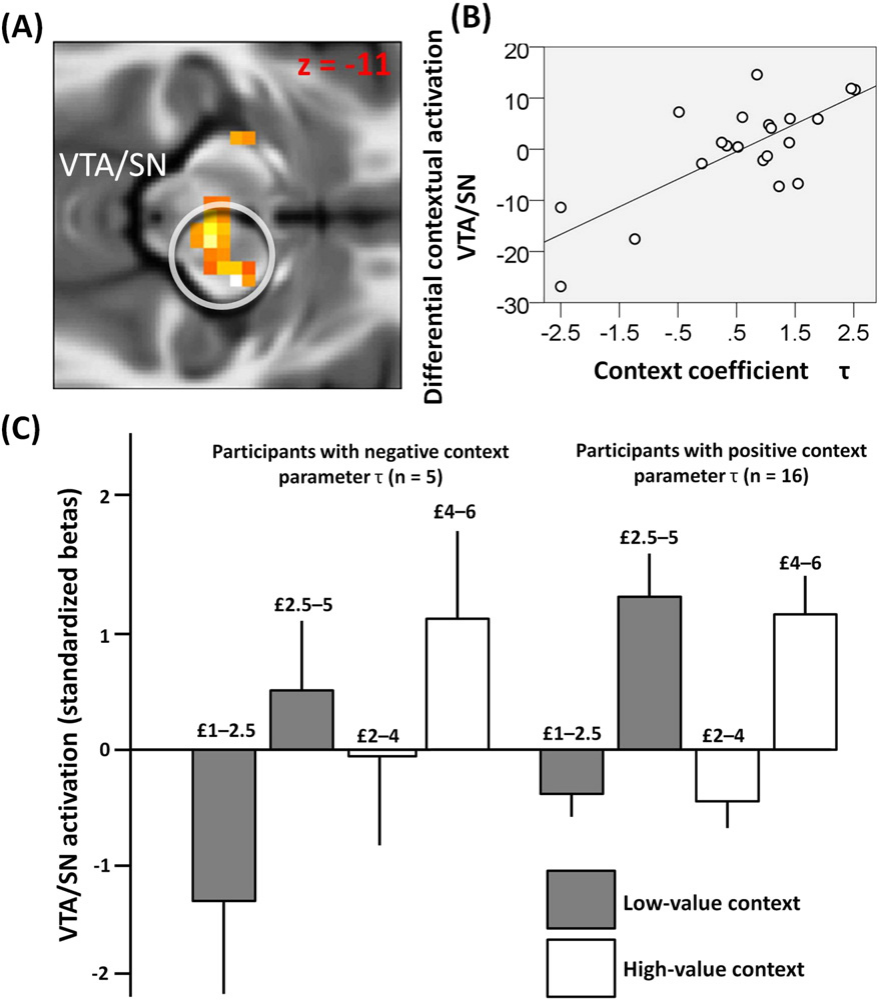
A: Activation in right VTA/SN showing, at option presentation, a correlation between the context coefficient τ (implementing a context effect by representing a parameter subtracted from the amount of the certain option in the high-value context) and a neural response for the contrast of low-value minus high-value context across all amounts. B: Results from this analysis are plotted for the right VTA/SN peak voxel (15, − 16, − 11; Z = 4.23, p < 0.001 SVC). Note that this graph is solely for the purposes of display; no further statistical analysis is conducted on it. C: VTA/SN activation (beta weights are standardized for each subject computing z-scores using the individual mean and standard deviation) as a function of monetary amount, separately for participants with negative (left, n = 5) and positive (right, n = 16) context parameter τ. Amounts are organized in two bins separately for each context. Activations are displayed for the peak VTA/SN voxel. Note that this graph is solely for the purposes of display; no statistical analysis is conducted on it.

We next investigated the relationship between a behavioural and neural adaptation to context. Substantial evidence indicates that an RPE response in VTA/SN and VST adapts to a contextual reward distribution (Park, S. Q., et al., 2012, Tobler, P. N., et al., 2005). However, from this previous literature it remains unclear whether this neural adaptation is connected with behaviour. Here we hypothesized that a behavioural context effect would be reflected in a reward adaptation expressed in VST and VTA/SN. In our task larger amounts of money, on average, were available in the high- compared to the low-value context. Therefore, we predicted that at the time of option presentation, context-insensitive participants should show a greater response in VST and VTA/SN within a high compared to low-value context. Conversely, context-sensitive participants who engage in more reward adaptation, should exhibit more similar VST and VTA/SN activation in the two contexts. To test these predictions, we focused on VST and VTA/SN voxels where activation correlated with the average subjective value across options in the very first analysis (at an uncorrected threshold of p < 0.05; SVC was performed on these voxels alone). We estimated a second GLM that included, for each context, four regressors associated with bins of increasing monetary amount (i.e., the vector [1 2 3 4]). Consistent with our prediction, we found that at option presentation a differential activation (across all amounts) in right VTA/SN contrasting the low-value minus high-value context significantly correlated with the context coefficient τ (15, − 16, − 11; Z = 4.23, p < 0.001 SVC; Fig. 3). However, no such correlation was found in left VTA/SN and VST.

**Fig. 4:**
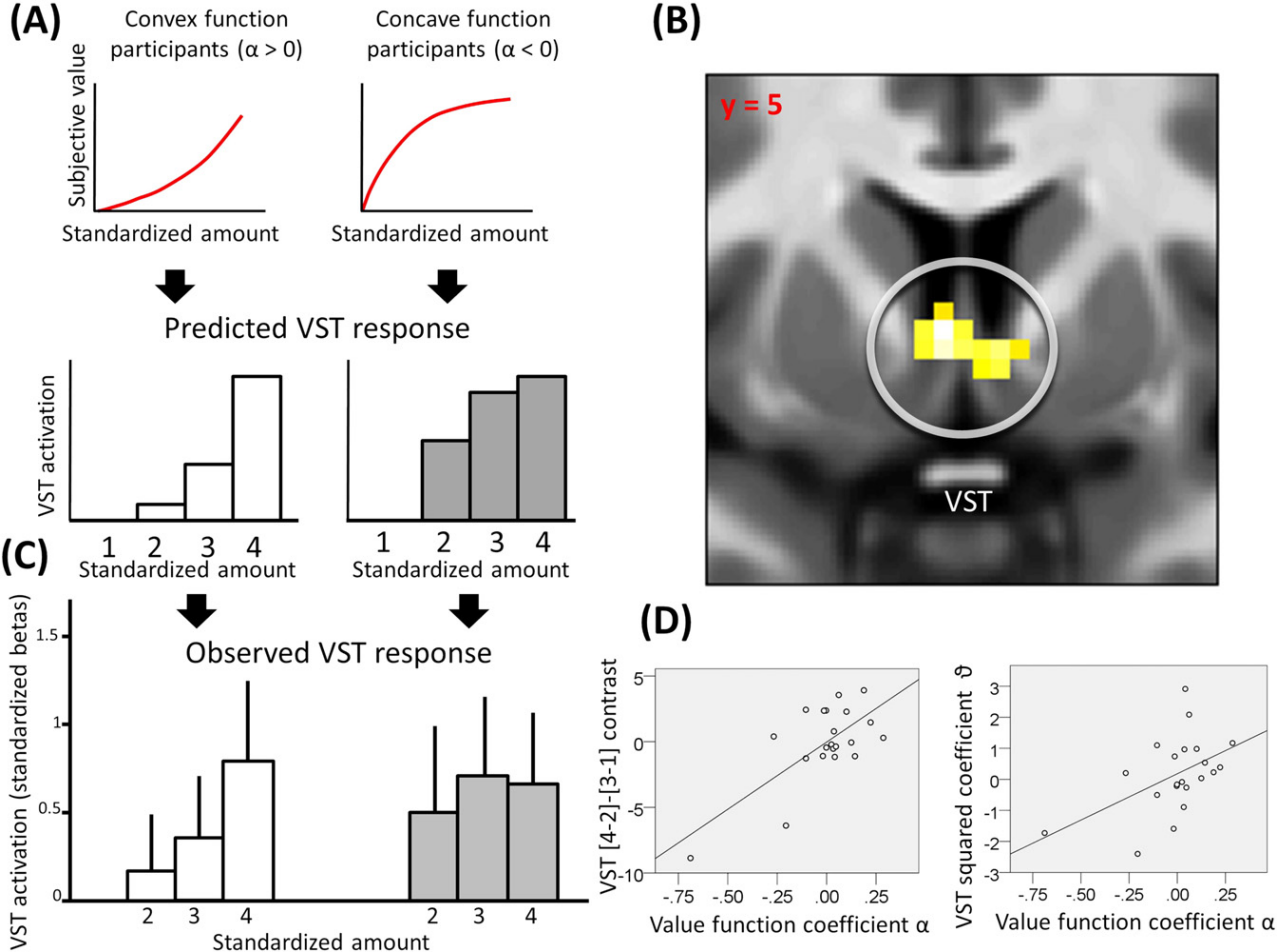
A: For convex (on the right, α > 0) and concave (on the left, α < 0) value function participants, predicted VST activation as a function of monetary amount, represented as four bins normalized across contexts. B: VST activation (right: 6, 5, − 2; Z = 3.02, p = 0.025 SVC; left: -3, 5, 1, Z = 3.53, p = 0.005 SVC) for the correlation between the coefficient α, determining the concavity or convexity of the individual subjective value function, and the contrast [4–2]-[3–1]. C: Observed VST activation (beta weights are standardized for each subject computing z-scores using the individual mean and standard deviation; the standardized beta associated with standardized amount = 1 is next subtracted to all other standardized betas) as a function of monetary amount, for concave (α < 0) and convex (α > 0) value function participants (error bars indicate standard errors). Activations are displayed for the peak VST voxel. Concave and convex subjective value functions estimated from behaviour were associated respectively with concave and convex neural responses with increasing amounts. D: On left, relationship between the coefficient α and the contrast [4–2]-[3–1] for the peak VST voxel (− 3, 5, 1, Z = 3.53, p = 0.005 SVC); on right, relationship between the behavioural value function coefficient α and the coefficient ϑ, corresponding to the second-order coefficient of a polynomial function fitted to the peak VST response (standardized betas) with different amounts (r(21) = 0.49, p = 0.023). Note these correlations remain statistically significant when neural data are transformed according to a square root transformation, rendering the analysis less affected by outliers (r = 0.561, p = 0.012 for the analysis correlating the value function coefficient α and the contrast [4–2]-[3–1] in ventral striatum; r = 0.508, p = 0.022 for the analysis correlating the value function coefficient α and the quadratic component of the ventral striatal response). These graphs are solely for the purposes of display; no further statistical analysis has been conducted on them.

We next tested whether a VST response to different levels of trial amount/variance reflected a non-linear subjective value function akin to that predicted by economic theories (D'Acremont, M. and Bossaerts, P., 2008, Kahneman, D. and Tversky, A., 1979, Schultz, W., et al., 2008, von Neumann, J. and Morgenstern, O, 1944). We focused on VST voxels where activation correlated with the average subjective value across options in the very first analysis (at an uncorrected threshold of p < 0.05; SVC refers to these voxels alone). We then considered the subject-specific non-linearity of the mapping from reward amounts/variances to subjective values. Fig. 4A shows how this is captured for four rewards of increasing amount (normalized across contexts) by the value function parameter α, with a clear neural difference for participants who had concave (value function parameter α < 0) and convex (α > 0) value functions, as derived from their choice behaviour. We examined these non-linearities of subjective value coding in the VST in a two-step analysis using the same GLM as for the context effect analysis. For each context, this included four regressors associated with bins of increasing amount (i.e., the vector [1 2 3 4]; see Fig. 4A). As a first step, we computed the contrast [4–2]–[3–1] that is independent of any correlation with objective monetary amount. Under the hypothesis that VST activity covaried with the subjective value, we expected this contrast to be positive and negative for subjects with behaviourally estimated convex and concave functions, respectively. Consistent with this, we found a significant correlation with the value function coefficient α in bilateral VST (right: 6, 5, − 2; Z = 3.02, p = 0.025 SVC; left: -3, 5, 1, Z = 3.53, p = 0.005 SVC; Figs. 4B–D; this result remained statistically significant when neural data were transformed according to a commonly used square root transformation, which is less affected by outliers (r = 0.561, p = 0.012)). As a second step of the analysis, we extracted the weights (i.e., the beta regressor coefficients) from the peak-activation voxel within this region of the four monetary amount-related bins and standardized these for each subject by computing z-scores with respect to the individual means and standard deviations. Then, in each subject, we estimated the second-order (i.e., quadratic) coefficient ϑ of a polynomial function mapping the amount-related bins (i.e., the vector [1 2 3 4]) to the corresponding standardized weights associated with VST activation. The quadratic coefficient ϑ was positively correlated with the behavioural value function parameter α (r(21) = 0.49, p = 0.023; Fig 4D right; this result remained statistically significant when neural data were transformed according to a commonly used square root transformation, which is less affected by outliers (r = 0.508, p = 0.022)). Altogether, these results support the idea that VST response to different levels of amount/variance can be characterized by a non-linear function estimated from behaviour.

## Discussion

It is well-established that decision-making is context-dependent (e.g., De Martino, B., et al., 2006, Guitart-Masip, M., et al., 2010, Huber, J., et al., 1982, Kahneman, D. and Tversky, A., 1979, Kolling, N., et al., 2014, Ludvig, E. A., et al., 2013, Stewart, N., et al., 2003, Tversky, A. and Shafir, E., 1992, Wright, N. D., et al., 2012, Wright, N. D., et al., 2013), though the specific mechanisms for this influence remain unclear. One possibility is that choices are influenced because subjective values are rescaled with respect to the contextual reward distribution so that they decrease and increase in high and low-value contexts respectively (Ludvig, E. A., et al., 2013, Stewart, N., et al., 2003, Stewart, N., et al., 2006). An alternative idea is that the prevailing contextual reward distribution is to some extent assimilated with objective reward values which would therefore subjectively increase and decrease in high and low-value contexts respectively (Seymour and McClure 2008). We compared these possibilities in a risky decision-making task in which participants chose between a certain monetary gain and a gamble associated with equal probability of getting either double that gain or zero. Crucially, the reward distribution varied across blocks such that, in different blocks, trial rewards were picked from one of two different, but partially overlapping, distributions.

We showed that whether participants gambled more or less for equivalent choices depended on participants' specific gambling preferences for different reward amounts. Participants who gambled more for larger amounts also risked more when equivalent choices were relatively larger within the context, while participants who gambled more for smaller amounts also risked more when equivalent choices were relatively smaller within the context. This finding is at odds with a proposal that subjective values are assimilated to the contextual reward distribution (Seymour and McClure 2008). Neither is it well explained by the original proposal of Prospect Theory (Kahneman and Tversky 1979), which does not consider context effects beyond those emerging from framing choices in terms of losses or gains (note that here all choices were in the gain domain). These data are not easily explained by Prospect Theory even if the safe amount is conceived as reference point. Indeed, in this instance prospect theory would predict that, due to loss aversion, risk aversion would increase with trial amount for all participants. This does not correspond to the pattern seen in the data which show individual differences in baseline gambling and individual differences in the preference to gamble with increasing trial amount.

Our results are consistent with the idea that subjective values are rescaled with respect to the contextual reward distribution. This is in keeping with a previous report regarding simultaneously presented options (Stewart et al. 2003) which we extend here into the temporal domain by showing a value rescaling affected by the distribution of reward within the temporal context (see also Ludvig et al., 2013). This is important because such a temporal factor is ubiquitous in ecological contexts (Stewart et al. 2006).

fMRI enabled us to examine the link between behavioural and neural adaptation to context. Our focus on VTA/SN was motivated by evidence indicating that a RPE signal in this area adapts to the current reward expectancy (Tobler et al. 2005). However, previous literature did not resolve whether the neural adaptation in VTA/SN is linked with contextual behavioural effects. At the time of option presentation, context-insensitive participants showed an increased VTA/SN response in the high-value compared to low-value context, while context-sensitive participants did not. This observation highlights a link between subjective value rescaling to the contextual reward distribution (as inferred from behaviour) and brain response adaptation in VTA/SN. Though caution needs to be exercised in making causal inferences from fMRI data, the results are consistent with a proposal that a VTA/SN reward adaptation process mediates an influence of context on risk preference. One potential mechanism underlying the effects we report derives from a theoretical proposal that tonic VTA/SN activity encodes an average reward representation (Niv et al. 2007) which might operate as a reference point against which option values are compared. Within this framework, our data indicate that an individual sensitivity to changes in tonic VTA/SN activity could influence learning of average reward representations that in turn determines neural and behavioural adaptation to context. Another possible mechanism is that the contextual reward statistics are processed in areas involved in representing more abstract contextual information, for example in the hippocampus and parahippocampal gyrus (Aminoff, E. M., et al., 2013, Holland, P. C. and Bouton, M. E., 1999), which would in turn modulate a response of neurons in VTA/SN according to the reward context. A recent study from our lab has reported contextual modulation for pain evaluation in orbitofrontal cortex (Winston et al., 2014). Although our main focus here was on VTA/SN and ventral striatum in the context of reward, investigating the relationship between VTA/SN and orbitofrontal cortex in both reward and punishment contextual adaptation is an important question for future research.

The nature of our design meant we were able to dissociate a baseline gambling tendency from a preference to gamble for large or small reward amounts/variances. These two components had an independent influence on behaviour, a novel finding at odds with some influential theories (D'Acremont, M. and Bossaerts, P., 2008, Kahneman, D. and Tversky, A., 1979, Schultz, W., et al., 2008, von Neumann, J. and Morgenstern, O, 1944) because it implies that a single factor, such as a value function or a variance sensitivity, is insufficient to capture risk preferences. A baseline gambling tendency might depend on a specific preference towards choosing a certain rather than a risky option, possibly related to psychological constructs such as novelty seeking (Cloninger, C. R., 1985, Friston, K., et al., 2013).

Our design also permitted a test of a hypothesis that the VST response to different levels of reward amount/variance could be characterized by a non-linear function akin to that proposed by economic theories (D'Acremont, M. and Bossaerts, P., 2008, Kahneman, D. and Tversky, A., 1979, Schultz, W., et al., 2008, von Neumann, J. and Morgenstern, O, 1944). Contrary to two previous studies (Christopoulos, G. I., et al., 2009, Niv, Y., et al., 2012), we found evidence supporting this hypothesis. One previous study (Levy et al. 2010) has reported that VST response to different reward amounts can be described with a non-linear function, but this fit was not significantly better than the fit for a linear function, thus rendering these prior results inconclusive on this point. Several characteristics also distinguish our study from that of Niv et al. (2012), including the fact that in the former gamble probabilities were learned rather than instructed (Hertwig and Erev 2009) and the inclusion of trials with no decision in the analysis (as opposed to choice trials). Furthermore, an amount-dependent risk preference was not estimated separately from a baseline gambling tendency, a procedure that might add noise or bias to the behavioural risk parameter estimation when these two variables are uncorrelated, as indeed we observed here. Our paradigm is also distinct from Christopoulos et al. (2009) who found no difference in VST activation for gamble choices associated with equivalent average magnitudes but different subjective values. In this study the procedure adopted might conceivably have increased attention towards the certain option and, together with evidence showing that attention influences VST value responses (FitzGerald et al. 2014), this might explain the observation that VST encoded the subjective value of the certain option alone. Indeed, in the former study the certain option varied more frequently than the gamble, and varied according to a partially predictable staircase procedure that might also have induced a motivation for subjects to predict future certain options. Our data better fit with the general proposal that VST represents subjective rather than objective value (Hsu, M., et al., 2009, Kable, J. W. and Glimcher, P. W., 2007, Niv, Y., et al., 2012, Pine, A., et al., 2009, Tom, S. M., et al., 2007).

At least two kinds of neural computations might underlie a preference to gamble more with large or small amounts, namely an implementation of a function mapping of reward amount to subjective value (Kahneman, D. and Tversky, A., 1979, von Neumann, J. and Morgenstern, O, 1944, Kable, J. W. and Glimcher, P. W., 2009, Padoa-Schioppa, C. and Assad, J. A., 2006), or an integration of information about reward amount and risk associated with measures of reward variability such as variance (D'Acremont, M. and Bossaerts, P., 2008, Preuschoff, K., et al., 2006, Schultz, W., et al., 2008). Our task cannot dissociate these two mechanisms since variance perfectly correlated with monetary amount. This consideration also affects the interpretation of subjective value computations in VST. It is important to stress that the response we observed in this region is inconsistent with an encoding of subjective value associated with variance alone, as this hypothesis predicts a decreased response with larger monetary amounts in subjects with a negative value function coefficient α. In fact we observed an increase with larger monetary amounts in all subjects, a finding in line with previous evidence (e.g., Christopoulos, G. I., et al., 2009, Niv, Y., et al., 2012, O'Doherty, J. P., et al., 2003, O'Doherty, J., et al., 2004).

The specific nature of signalling in VST at option presentation is unclear, with candidate variables including average subjective value across options and subjective value of the chosen option. These variables co-varied systematically across our participants rendering the current data uninformative on this issue. However, our test of a non-linear function mapping reward amount/variance to VST activity is not affected by whether VST signals average value, the value of the chosen option, or both. Notably we also observed a response in VST that correlated with the subjective value difference between the chosen and unchosen option, which in our task was orthogonal across subjects with respect to the average value across options. This suggests that VST might play a role in value comparison across options. Despite a tight coupling between VTA/SN and VST, we found evidence of contextual adaptation in the former but not in the latter region. Though absence of evidence should be considered weak evidence of absence, future research should also explore the hypothesis that these two regions might operate according to different reference points.

Substantial human and non-human evidence has highlighted an important role for vmPFC in value-based choice (Bartra, O., et al., 2013, Blair, K., et al., 2006, Boorman, E. D., et al., 2009, FitzGerald, T. H., et al., 2009, Plassmann, H., et al., 2007, Strait, C. E., et al., 2014, Tom, S. M., et al., 2007). However, what variable is represented in this area during choice behaviour remains contentious. A subjective value difference between the chosen and unchosen option (Boorman, E. D., et al., 2009, FitzGerald, T. H., et al., 2009, Hunt, L. T., et al., 2012, Strait, C. E., et al., 2014) and the average subjective value across options (Blair, K., et al., 2006, Hunt, L. T., et al., 2012) are two candidate quantities. However, these are highly correlated in many previous designs. In our task design, we could decorrelate these quantities since the average subjective value depended largely on the trial monetary amount that was the same for all participants, whereas the subjective value difference between chosen and unchosen option depended on individual preferences. While we found a vmPFC signal correlating with the latter variable, we did not find any relationship with the former variable. A recent study on value-based choice with magnetoencaphalography (MEG) has reported an early vmPFC signal correlating with average value after option presentation, followed by a later signal correlating with value difference between chosen and unchosen option (Hunt et al. 2012). An advantage of our design is that it allowed us to decorrelate these two measures. However, an apparent discrepancy between the two studies might be explained by several factors, including the possibility that an early vmPFC response to average value has only a marginal impact on BOLD signal. In addition, in our task the monetary amount alone is a salient feature, whereas the task used in Hunt et al. (2012) involves both reward amount and probability. This difference might explain a discrepancy between the two studies coupled with the fact that vmPFC response to average subjective value across options might emerge only when different dimensions need to be integrated. It is noteworthy that vmPFC was systematically more active during certain compared to risky choices. This hints that vmPFC activity might be biased towards representing safe choices independent of considerations related to individual risk preferences.

In line with substantial evidence indicating that activity in anterior insula correlates with stimulus salience, and therefore with subjective value in appetitive domains (Bartra et al. 2013), we observed an association between a response in this region and average subjective value across options. Data from a passive risk-return task also indicate that activation in anterior insula increases with the entropy of the distribution of rewards (Critchley, H. D., et al., 2001, Preuschoff, K., et al., 2008, Rudorf, S., et al., 2012), a variable closely associated with risk. However, it is unclear whether such entropy-related responses are linked with choice. We observed that BOLD signal in insula was stronger when participants chose the gamble (associated with increased entropy) compared to when they chose the certain option. These results might appear inconsistent with a previous study showing insula response associated with switching from choosing risky to choosing safe options (Kuhnen and Knutson 2005). However, in this study, the history of reinforcement was relevant and therefore learning might have influenced the reported effect (Palminteri et al. 2012). We found no evidence of learning influences in our task, and hence our findings suggest that insula activity increases for choices of risky options, independent of any learning effect.

In sum, our findings show that choice behaviour adapts to the temporal contextual reward distribution and that VTA/SN response is linked with this adaptation process. This is in line with evidence that human preferences are often inconsistent across situations, but at the same time suggests such inconsistencies might be adaptive to environmental demands. This raises an intriguing possibility that syndromes characterized by dysfunctional decision-making, such as drug abuse and mood and anxiety disorders, might be linked to impairments in adapting choice strategies to context.

## Figures and Tables

**Table 1:** Comparison of behavioural models of choice behaviour. The first column reports the free parameters of each model. For some models, two different free parameters of the same kind were estimated, separated for block 1–2 and block 3–4 (e.g., μ_1–2_ and μ_3–4_, respectively) or for high-value and low-value context (e.g., μ_HV_ and μ_LV_, respectively). BIC was summed across participants.

Free parameters	BIC
Random	16245
μ	14481
α	14198
μ, α	12824
μ, α, τ	12665*
μ_1–2_, μ_3–4_, α, μ, τ	12675
μ_HV_, μ_LV_, α, μ, τ	12708
μ, α_1–2_, α_3–4_, τ	12688
μ, α_HV_, α_LV_, τ	12696
μ, α, τ_1–2_, τ_3–4_	12709
μ, α, τ (divisive normalization)	12840

**Table 2:** Relationship between variables related with value and risk that varied trial-by-trial and across subjects. For each subject, the values of the variables were estimated trial-by-trial using the computational model of behaviour and the individual parameters. The first column indicates pairs of variable. The second column indicates the number of participants for which the Pearson correlation between the pair of variables was statistically significant. The third and fourth columns report respectively the mean and standard deviation of the Pearson coefficient across participants. The fifth and sixth columns report respectively the t-statistic and the p value relative to the one sample t-test on the Pearson coefficients.

Variables	N participants	Mean (r)	SD (r)	t-statistic (r)	p value (r)
Value of chosen minus unchosen AND average value	18	− 0.05	0.47	− 0.47	p = 0.64
Average value AND gambling vs certain	14	0.12	0.32	1.72	p = 0.1
Value of chosen minus unchosen AND gambling vs certain	21	0.20	0.69	1.30	p = 0.21
Value of chosen minus unchosen AND value of chosen	16	0.36	0.42	3.97	p < 0.001
Average value AND value of chosen	21	0.79	0.48	7.44	p < 0.001
Gambling vs certain AND value of chosen	16	0.16	0.35	2.11	p = 0.05
