# Supplementary material for: The influence of contextual reward statistics on risk preference
Source: Neuroimage. 2016 Mar;128:74–84. doi: 10.1016/j.neuroimage.2015.12.016 (PMC4767216; doi:10.1016/j.neuroimage.2015.12.016)
Supplement: Supplementary file 1 — Supplementary material. [file mmc1.docx]

**SUPPLEMENTAL MATERIAL**

**Supplemental behavioural analyses**

Here, we report further behavioural analyses. The order of block presentation did not affect gambling percentage (F(3,17) = 0.04, p =0.99), the effect of monetary amount on gambling percentage (F(3,17) = 0.13, p = 0.94), the gambling percentage for overlapping amounts in the low-value minus high-value context (F(3,17) = 0.30, p = 0.83) and the corrected context effect (F(3,17) = 2.41, p = 0.1).

We next tested whether choice behaviour was stable along the task and found that gambling percentage did not change along blocks (F(3,60) = 0.22, p = 0.88). In addition, we correlated the average gambling percentage of each of the four blocks with each other and found a strong positive correlation in all cases (all r > 0.76, p < 0.001). We used logistic regression to estimate the effect of trial monetary amount on gambling percentage (i.e., individual slope parameters) separately for each of the four blocks and correlated them with each other, finding a positive correlation in all cases (all r > 0.80, p < 0.001). Finally, we computed the gambling percentage for overlapping amounts in the low-value minus high-value context separately in each of the two sessions and found that the two measures were positively correlated (r = 0.66, p = 0.001). Overall, these results show that individuals’ choice behaviour was stable during the task.

To test for the effect of other variables on choice behaviour, we estimated a set of logistic regression models, each including one of the following predictors (see supplementary tab. 1): pressing the same button as in previous trial; choice of gambling or certain option in previous trial; monetary amount in previous trial; RPE in previous trial. These models did not improve model fitting (estimated with the Bayes Information Criterion – BIC) compared to a simpler intercept model. The only variable that improved the model fitting was the monetary amount at the current trial.

For the model-based analysis based on subjective value estimate, note that the best-fitting model does not include a temperature parameter. Due to the structure of the task where options had equal average amount, $V_{\mathrm{GAMB}} - V_{\mathrm{CERT}} =x+ \alpha x^{2} -x+ \mu$ $=\alpha x^{2}+ \mu$, corresponds to a logistic regression model with α as scaling coefficient and where a second scaling coefficient like a temperature parameter would be redundant. Indeed, a model that included a temperature parameter had a higher BIC than the preferred model described in the main text (BIC = 12957). Estimation of model parameters was unbounded and performed using fminsearch Matlab function. Gambling probability as a function of monetary amount for different sets of parameters is shown in figure S1.

As expected, parameters of the winning model were correlated with behavioural measures such that the value function coefficient α correlated with the individual effect of amount on gambling percentage (i.e., individual slope parameters estimated with logistic regression; r(21) = 0.97, p < 0.001); the gambling bias μ correlated with the average gambling percentage (r(21) = 0.58; p = 0.006); the context coefficient τ correlated with the corrected context effect (r(21) = 0.45, p = 0.04). Across participants the context coefficient τ was unrelated either to the value function parameter α (r(21) = 0.043, p =0.852) and the gambling bias μ (r(21) = 0.81, p = 0.727), while the latter two parameters were inversely correlated (r(21) = -0.680, p = 0.001). This correlation is not surprising given that the gambling bias parameter μ corresponds to the gambling probability associated with a gamble having an hypothetical variance of zero, and thus is expected to be smaller for positive than negative value function parameter α keeping the overall gambling percentage constant.

We also analyzed the effect of trial amount and gambling (a binary variable reflecting the choice of the gamble or of the safe option) on reaction times, and found no significant effect across participants (trial amount: t(20) = 0.72, p = 0.48; gambling: t(20) = 0.37, p = 0.72). In addition, the individual beta parameters linking trial amount to reaction time were unrelated with the beta parameters linking trial amount with choice (r(21) = -0.05, p = 0.83), and the beta linking gambling to reaction times were unrelated with the individual average gambling proportion (r(21) = 0.21, p = 0.36).

To test that mistakes did not occur systematically in one condition, for subjects who made at least two errors (n = 13), we tested a logistic regression model of errors/correct responses as function of trial amount and found no significant effect across subjects (r(13) = 0.19, p = 0.53).

**Supplemental analysis of vmPFC**

We performed further analyses on the nature of vmPFC value signalling. Behaviourally the subjective value of the chosen option and the average subjective value across options were significantly correlated in all participants. The direction of the correlation was systematically positive rendering the data unsuited to disentangle the effects of these two variables on brain activity (see Table 2 in main text).

Evidence suggests the value of the chosen option is another candidate variable associated with vmPFC activity (Kable & Glimcher, 2007), especially at a very late point during the decision process (Strait et al., 2014). The difference between chosen and unchosen option and the subjective value of the chosen option showed a correlation across participants (see Table 2 in main text). Therefore our behavioural data were non-optimal to disentangle the impact of these two variables on brain activity. However, when using a GLM equivalent to the first one described in Methods (see main text) except that the value difference between chosen and unchosen option was replaced by the value of the chosen option as parametric modulator (both estimated with the behavioural computational model), we found no vmPFC activity related to the value of the chosen option even at p < 0.05 uncorrected.

These data are more supportive of an encoding of the value difference between chosen and unchosen option rather than of the value of the chosen option. A possible explanation of the discrepancy between our results and previous studies (Kable & Glimcher, 2007; Strait et al., 2014) is that a value of the chosen option might affect BOLD signal in paradigms in which option presentation and action performance are delayed by design, an aspect absent in our task.

Kable, J. W., & Glimcher, P. W. (2007). The neural correlates of subjective value during intertemporal choice. *Nature neuroscience*, *10*(12), 1625-1633.

Strait, C. E., Blanchard, T. C., & Hayden, B. Y. (2014). Reward value comparison via mutual inhibition in ventromedial prefrontal cortex. *Neuron*, *82*(6), 1357-1366.


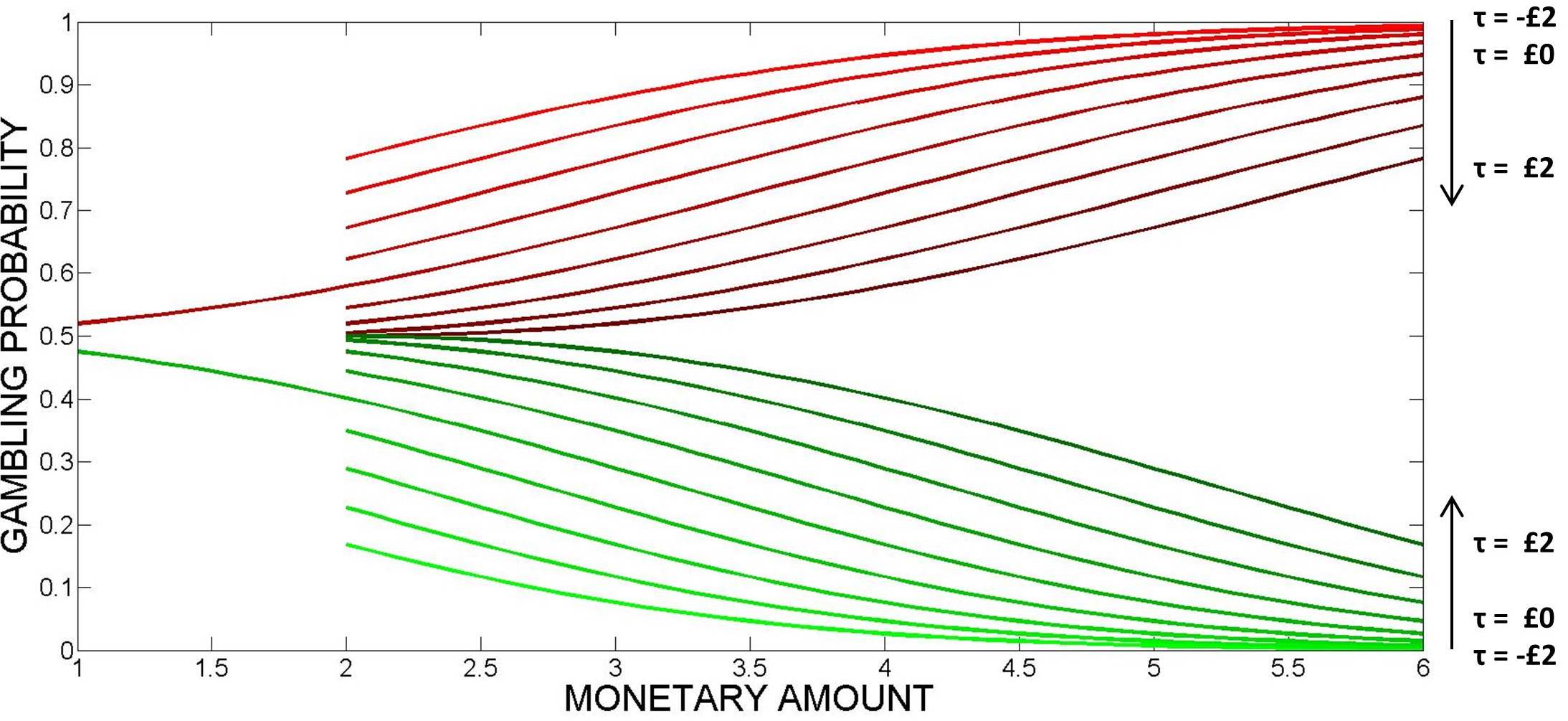


**Figure S1.** Plot of the gambling probability as a function of trial monetary amount for a set of simulated agents with specific parameters. Red lines represent agents with a positive value function coefficient α (equal to 0.08) and green lines represent agents with a negative value function coefficient α (equal to -0.1; these values correspond to median estimates from participants’ data). Behaviour of agents with τ equal to zero is represented by lines extending from £1 to £6. For the high-value context extending from £2 to £6, agents with different τ are plotted in which τ increases in £0.5 steps from -£2 to £2 along a bright-to-dark gradient.
